# Benign Intracranial Calcified Lesion or a So-Called Brain Stone: A Challenging Diagnosis

**DOI:** 10.7759/cureus.39596

**Published:** 2023-05-28

**Authors:** Kivanc Yangi, Ajlan Uzunkol, Suat Erol Celik

**Affiliations:** 1 Neurological Surgery, Prof. Dr. Cemil Tascioglu City Hospital, Istanbul, TUR

**Keywords:** capnon, arteriovenous malformations, cerebral cavernoma, brain stone, intracranial calcifications

## Abstract

Brain stone is an umbrella term for benign intracerebral calcifications and may be associated with various diagnoses. The surgical decision should be made on a case-by-case basis. Sometimes, conservative management should be considered, irrespective of the underlying pathology. We present a critical case with a brain stone treated conservatively. A 17-year-old female patient was admitted to our department with a headache. The neurological examination revealed no abnormal findings. Cranial CT and MRI scans showed a contrast-enhanced, highly calcified lesion located deep in the white matter at the level of the left centrum semiovale. Surgery was found unnecessary. The patient presented no neurologic deficits or symptoms during the three-year follow-up period. In this case, the differential diagnosis included arteriovenous malformations (AVMs), cavernomas, calcifying pseudoneoplasms of the neuroaxis (CAPNON), etc. The localization of the lesion, expression of the symptoms, and potential outcomes of a possible surgery should be carefully estimated before making the final decision. In summary, conservative treatment should also be considered for critically located, benign calcified lesions, irrespective of pathology, unless they cause intense neurologic symptoms or deficits.

## Introduction

Benign intracranial calcifications are relatively common findings in various pathologies. They are also called. *cerebral calculus* or *brain stone*. Brain stone is an umbrella term for "large, solitary or multiple, well-circumscribed, hard bony areas of pathologic intracerebral calcification" that represent the final stage in the evolution of specific non-neoplastic space-occupying lesions [1]. Brain stones may be located intra-axially or extra-axially. Although extra-axial brain stones are usually tumors or physiologic calcifications, intra-axial brain stones are generally associated with infections; congenital, metabolic, or endocrine anomalies; vascular pathologies; and neoplasms [2,3]. Sometimes, there is no need to clarify the pathology beneath the brain stone to decide the case. Conservative management may be enough for these patients, irrespective of pathology. Close clinical follow-up might be enough if there are no neurologic deficits. We present the case of a 17-year-old female patient with a highly-calcified lesion extending from the level of corona radiata to the lateral ventricle on the left side.

## Case presentation

**Figure 1:**
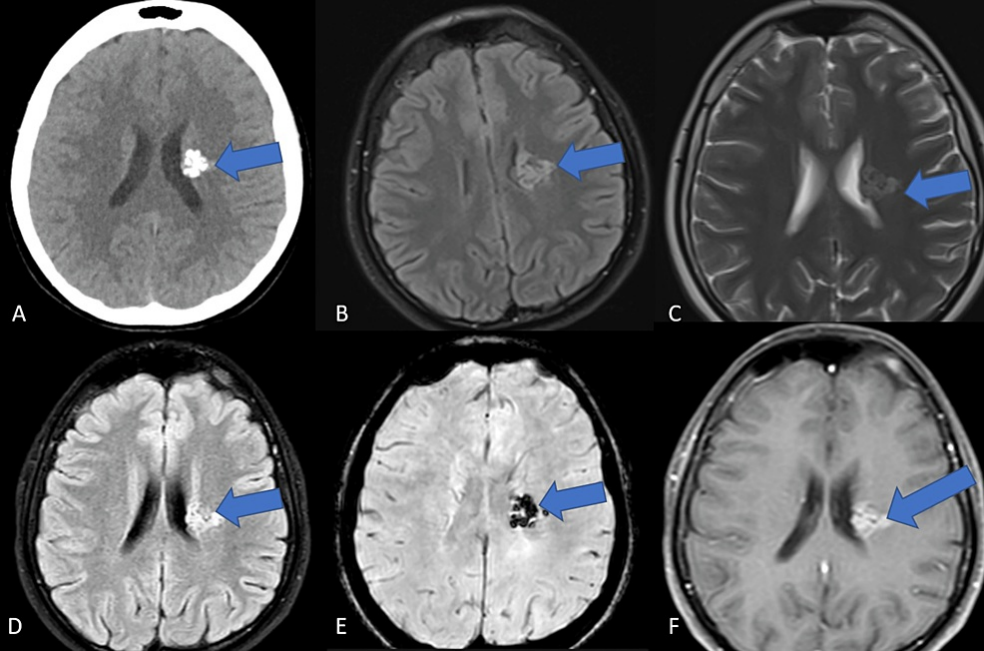
Radiological imaging of the brain stone A: Non-contrast enhanced cranial CT showing a hyperdense lobulated space-occupying lesion with multiple calcifications, 25x30 mm in diameter, at the level of left centrum semiovale; suspected radiological diagnosis is cavernoma (blue arrow) B: T1-weighted non-contrast-enhanced cranial MRI showing an iso-hyperintense lesion, approximately 25x30 mm in diameter, containing suspected vascular structures with irregular borders; suspected radiological diagnosis is a possible arterio-venous malformation (blue arrow) C: T2-weighted cranial MRI indicating an iso-hyperintense lesion at the level of left corona radiata with central hypodensities, and no perilesional edema observed (blue arrow) D: Cranial fluid attenuated-inversion recovery (FLAIR) MRI indicating a hyperintense calcified space-occupying lesion, 25x30 mm in diameter, next to the left lateral ventricle; suspected radiological diagnoses are cavernoma and cavernous angioma (blue arrow) E: Susceptibility-weighted cranial (SWI) MRI) scan indicating the calcified lesion; radiological differential diagnosis includes ganglioglioma, oligodendroglioma, and ependymoma because of intense calcifications (blue arrow) F: T1-weighted contrast-enhanced cranial MRI showing heterogeneously contrast-enhanced, highly calcified, lobulated space-occupying lesion at the level of corona radiata, next to the left lateral ventricle (blue arrow)

A 17-year-old female patient with no medical history was admitted to our outpatient clinics with a headache. She was experiencing a dull headache that had worsened over the past two months and was more intense at night. The patient had no pertinent family history of systemic or neurologic disease and showed no impairment in social functioning. The patient is a non-smoker and does not use illicit or recreational drugs. The neurologic examination was unremarkable. Computed tomography revealed a left frontoparietal calcified and lobulated space-occupying lesion at the level of the centrum semiovale, located deep in the white matter. These findings were consistent with cavernoma, as per radiologists (Figure 1).

Subsequent diffusion-MRI and contrast-enhanced MRI scans were gathered. Diffusion restriction was not observed. According to the MRI scans, tumors were thought to include vascular structures. These findings were interpreted to be consistent with arteriovenous malformations (AVMs). Contrast-enhanced MRI revealed a highly contrast-enhanced space-occupying lesion, 25x30 mm in diameter, extending from the corona radiata to the lateral ventricle on the left side, including vascular structures. This image was thought to be consistent with a low-grade glioma. The lesion was iso-hyperintense in T1-weighted non-contrast-enhanced sequences (Figure 1).

After discussing vascular pathologies, the CT-angiography results were found to be consistent with a calcified cavernoma. Two months later, since there was no consensus on the initial diagnosis, a second contrast-enhanced MRI and MRI spectroscopy were performed. The MRI spectroscopy was unsuccessful because of the lesion’s calcified nature. Ganglioglioma, oligodendroglioma, and ependymoma were considered in the differential diagnosis by the second center’s radiologists, but not the cavernous angioma because of the lesion’s intense contrast enhancement. A further two months later, contrast-enhanced MRI scans were performed in a third, different center. This MRI showed an intra-axial, heterogenous contrast-enhanced lesion, 25x30 mm in diameter, just like the other MRI scans. There was no perilesional edema or mass effect of the lesion reported. The lesion was iso-hyperintense on T1-weighted non-contrast enhanced sequences and hyperintense on T2-weighted non-contrast enhanced sequences. The lesion was thought to be consistent with intense cerebral calcifications (cerebral calculi or brain stones); oligodendrogliomas, AVMs, and calcifying pseudoneoplasms of neuroaxis (CAPNON) were considered in the differential diagnosis.

Although there was no consensus on the initial diagnosis, the lesion was considered benign. And so, surgery was deferred. The patient was followed up. Three months later, at the first follow-up, the CT scan showed no increase in the size and diameter of the lesion. Painkiller medications were prescribed, and the patient declared she was headache-free. The patient showed no further headaches in the three-year follow-up period. The patient continues to be followed up at regular intervals.

## Discussion

Brain stones are intracranial calcifications associated with a broad range of diagnoses. Patients with brain stones may present with seizures or be discovered incidentally on imaging [3,4]. They can be located intra- or extra-axially. As Gezercan et al. said, lesions >1 cm may be classified as brain stones, while lesions <1 cm may be classified as calcifications [3].

Cavernomas and AVMs are the most common causes of these intracranial calcifications. Large calcified intracranial lesions, or brain stones, are common pathologies. A few reports in the literature used the term ''brain stone'' and explained its association with various intracranial lesions [3]. In our case, a 17-year-old female patient presented with an intracranial calcified lesion. No consensus was reached on the initial diagnosis; it may be associated with calcified tumors, AVMs, or calcified cavernomas. Regardless of the pathology underneath, since the patient showed no neurological abnormalities or seizures and no increase in the size or diameter of the lesion, the most appropriate treatment method was conservative treatment. The decision to undertake or defer surgery should be considered when assessing benign intracranial calcified lesions. However, the lesion localization, expression of the symptoms, and potential outcomes of a possible surgery should be carefully estimated before the final decision. In this case, the definitive diagnosis would be a cavernoma, but it would not change our approach to the patient.

When considering intra-axial brain stones, neoplastic, infectious, congenital, and metabolic causes should also be considered [2]. Different tumors may cause intracranial calcifications. The blood supply can become insufficient when the tumor rapidly grows and necrosis starts. This condition can disrupt calcium metabolism and result in calcium deposition [5-7]. Oligodendrogliomas are among the most commonly calcified brain tumors, mostly associated with brain stones [2]. In our case, the pathology behind the lesion would also be a tumor, but it is most likely a vascular lesion because of its vascularization, shape, and heterogeneous contrast enhancement. To summarize, a definitive diagnosis of brain stones is only sometimes necessary. The most crucial point in these cases is how to approach a patient with a brain stone. Our study emphasizes the importance of a conservative approach rather than interventional approaches such as surgery or biopsy.

## Conclusions

Benign intracranial calcifications may be associated with a wide range of diagnoses. Although finding the pathology of the lesion is crucial, a definitive diagnosis may not be necessary for surgical decision-making in such cases. Although there was no consensus on a definitive initial diagnosis, the lesion was considered benign regardless of pathology. Because of the benign-looking lesion, its critical localization, and the patient's intact neuro-exam, surgery was found unnecessary, and the patient was followed up. During the three-year follow-up period, the patient showed no more headaches. Since only a few cases are reported in the literature using the term brain stone, more studies are needed to clarify the term and compare the approaches to these lesions.
